# Disulfidptosis ‒ related lncRNAs are biomarkers of prognosis and immune response in Head and Neck Squamous Cell Carcinoma

**DOI:** 10.1016/j.bjorl.2025.101625

**Published:** 2025-05-15

**Authors:** Ruilin Wang, Qi Zhang, Yuxiu Ma, Xuelin Liu, Tian Lan, Hongling Li

**Affiliations:** a Gansu University of Traditional Chinese Medicine, First Clinical Medical College, Lanzhou, Gansu, China; b Gansu Provincial Hospital, Department of Oncology, Lanzhou, Gansu, China

**Keywords:** Head and Neck Squamous Cell Carcinoma, Disulfidptosis, Biomarkers, Prognosis, lncRNA

## Abstract

**Objective:**

This study aims to explore the role of Disulfidptosis-Related long Non-Coding RNAs (DRlncRNAs) in the prognosis and immune infiltration of Head and Neck Squamous Cell Carcinoma (HNSCC).

**Methods:**

Using bioinformatics approaches, this study investigates the prognostic significance of DRlncRNAs in HNSCC patients and their potential association with the immune microenvironment. RNA sequencing data and clinical information for HNSCC were obtained from The Cancer Genome Atlas (TCGA) database. DRlncRNAs were identified through Pearson correlation analysis, and a prognostic model consisting of six DRlncRNAs was constructed using Least Absolute Shrinkage and Selection Operator (LASSO) regression, along with univariate and multivariate Cox analyses.

**Results:**

The predictive performance of the model was assessed using Receiver Operating Characteristic (ROC) curves and Principal Component Analysis (PCA), and further validated using calibration curves, a nomogram, and univariate/multivariate Cox analyses. In addition to functional enrichment analysis, the associations between the model and Tumor Mutation Burden (TMB), immune cell infiltration, and drug sensitivity were also examined.

**Conclusion:**

We developed a novel predictive model composed of six DRlncRNAs to predict the prognosis of HNSCC patients and proposed potential clinical therapeutic targets from the perspective of disulfidptosis.

**Level of evidence:**

Level 5.

## Highlights

• Disulfidptosis-related lncRNAs can predict the prognosis of HNSCC patients.
• Disulfidptosis-related lncRNAs are associated with tumor mutational burden in HNSCC.
• Disulfidptosis-related lncRNAs are closely related to immune responses in HNSCC.

## Introduction

Head and Neck Squamous Cell Carcinoma (HNSCC), originating from the mucosal epithelium of the oral cavity, pharynx, and larynx, is the most common malignant tumor in the head and neck region.^1^ HNSCC is the seventh most common cancer worldwide, posing a serious threat to human health. In 2020, there were 931,922 new cases and 467,125 deaths attributed to HNSCC, and its incidence is expected to continue rising. By 2030, the number of HNSCC patients is projected to increase by 30%.^2^ Smoking, alcohol consumption, high-risk Human Papillomavirus (HPV), and Epstein-Barr Virus (EBV) infections are commonly recognized as risk factors for HNSCC.^3^, ^4^, ^5^ For locally or regionally confined HNSCC, primary curative treatment strategies include surgical resection, radiotherapy, and systemic therapy.^1^ In patients with recurrent or metastatic HNSCC, some may achieve a cure through salvage surgery, re-irradiation, or metastatic lesion resection, while others are considered for systemic treatment.^6^, ^7^ Despite extensive research on HNSCC over the past decades, its prognosis remains poor. Notably, the 5-year survival rate for HPV-negative HNSCC patients is only 40%‒60%, and for 30%‒50% of advanced-stage patients who develop local or regional recurrence, the 2-year survival rate is even lower.^8^ Therefore, it is even more crucial to develop a prognostic model for HPV-negative HNSCC patients and explore potential therapeutic targets.

Liu et al. discovered that cystine uptake mediated by SLC7A11 and its reduction to cysteine is highly dependent on Nicotinamide Adenine Dinucleotide Phosphate (NADPH) produced through the glucose-pentose phosphate pathway. Therefore, under glucose starvation, NADPH is rapidly depleted in SLC7A11-high-expressing cells, leading to the abnormal accumulation of cystine and other disulfides, which triggers disulfide stress and causes rapid cell death.^9^ Further studies revealed that under glucose starvation, the combination of high cystine uptake and insufficient NADPH supply in SLC7A11-high-expressing cells results in NADPH depletion, abnormal disulfide bond formation in actin cytoskeletal proteins, actin network collapse, and subsequent cell death. This novel form of cell death cannot be inhibited by conventional cell death inhibitors or prevented by knocking out ferroptosis/apoptosis-related key genes. Moreover, thiol-based oxidants can significantly enhance this type of cell death, suggesting that it does not belong to any known cell death type. Consequently, it has been named disulfidptosis.^10^ Although the relationship between disulfidptosis and HNSCC has not been thoroughly investigated, based on the mechanism of disulfidptosis, it is speculated that it may play a role in the occurrence and progression of HNSCC.

Long non-coding RNAs (lncRNAs) are a class of RNA molecules characterized by two distinct features: a length of over 200 nucleotides and a lack of protein-coding ability.^11^ In the diagnosis and prognosis of HNSCC, lncRNAs are considered promising tumor biomarkers. This is partly due to their frequently dysregulated expression in HNSCC patients and partly because they can be easily detected in tissues and serum.^12^ Several prognostic models have already been developed to predict the outcomes of HNSCC patients, including models based on ferroptosis-related lncRNAs,^13^ cuproptosis-related lncRNAs,^14^ and N6-methyladenosine (m6A)-related lncRNAs.^15^

However, the role of Disulfidptosis-related lncRNAs (DRlncRNAs) in the prognosis of HNSCC patients remains to be explored. Therefore, we aim to investigate DRlncRNAs as biomarkers for HNSCC patients to evaluate their prognosis and provide potential therapeutic targets.

In this study, a predictive model was constructed using six DRlncRNAs, and its predictive capability was validated. Additionally, the relationships between the risk score and Tumor Mutation Burden (TMB), immune cell infiltration levels, and drug sensitivity were explored, aiming to evaluate the prognosis of HNSCC patients and identify potential therapeutic targets.

## Methods

### UALCAN database

The UALCAN database (https://ualcan.path.uab.edu/index.html) contains clinical data from TCGA, which can be used to analyze gene expression, DNA methylation, gene correlations, and survival outcomes in cancer patients. In this study, the database was utilized to investigate the expression of the SLC7A11 gene in samples from HNSCC patients and normal tissues.

### Data collection

In March 2023, clinical data (including survival time, survival status, age, sex, grade, and stage) and transcriptomic expression profiles were obtained from the TCGA website (https://portal.gdc.cancer.gov/) for 44 normal tissue samples and 521 HNSCC samples. Samples with incomplete survival information and normal tissue samples were further filtered, leaving a final selection of 518 HNSCC patients. Using the clinical information and related RNA-Seq data from these patients, the relationship between lncRNA expression characteristics and the prognosis of HNSCC patients was analyzed. Annotation information for lncRNAs was retrieved from the GENCODE website (https://www.gencodegenes.org/).

### Identification of disulfidptosis-related lncRNAs

Based on previously published literature, genes associated with disulfidptosis include NDUFS1, OXSM, LRPPRC, NDUFA11, NUBPL, NCKAP1, RPN1, GYS1, SLC3A2, and SLC7A11. Through co-expression analysis between these genes and lncRNAs, DRlncRNAs were identified using the “limma” package in *R* software (correlation coefficient > 0.4, *p* <  0.001).

### Construction of prognostic model

The “caret” package in *R* was used to randomly divide the samples into two groups in a 1:1 ratio, resulting in a training set (*n* = 259) and a testing set (*n* = 259). The training set was used to construct the DRlncRNAs model, while the testing set was used to validate the accuracy of the established model. Using the “glmnet” package in R, LASSO Cox regression analysis was performed to identify 14 DRlncRNAs associated with Overall Survival (OS) in HNSCC. Further multivariate Cox regression analysis narrowed down the selection to six DRlncRNAs (AC090587.1, EMSLR, AL590617.2, RAB11B-AS1, AP002478.1 and AC093827.4). Subsequently, the prognostic risk score was calculated using the following formula: $\sum_{i=1}^{n}{coef}_{i}*x_{i}$, where the standardized number of each DRlncRNA is represented by $x_{i}$ and the coefficient is represented by ${coef}_{i}$.By filtering HPV-negative samples, a prognostic prediction model specifically for HPV-negative HNSCC patients was further developed.

### Validation of the risk model

Using the median risk score as the cutoff, the samples were divided into low-risk and high-risk groups. Subsequently, Kaplan-Meier survival curves were applied to analyze the differences in OS between the high-risk and low-risk groups. The accuracy and diagnostic value of the DRlncRNAs were assessed by plotting the Receiver Operating Characteristic (ROC) curve and calculating the Area Under the Curve (AUC) using *R* packages. To further validate the risk model, Principal Component Analysis (PCA) was performed, and the results were visualized using the “scatterplot3D” tool in R software.

### Construction and calibration of the nomogram

Using the *R* packages “rms”, “regplot”, and “survival”, a nomogram was constructed based on patients’ risk scores and clinical information (tumor grade, tumor stage, gender, and age). The accuracy of the nomogram's predictions was assessed using calibration curves.

### Functional and pathway enrichment analysis

Under the criteria of log₂ |fold change| > 1 and False Discovery Rate (FDR < 0.05), the *R* package “limma” was used to identify differentially expressed genes between different risk groups. For functional and pathway enrichment analysis, as well as data visualization, the *R* packages “clusterProfiler”, “enrichplot”, “org.Hs.eg.db”, and “ggplot2” were utilized. These analyses were conducted based on the Gene Ontology (GO) and Kyoto Encyclopedia of Genes and Genomes (KEGG) databases.

### Assessment of tumor-infiltrating immune cells and immunotherapy

The ESTIMATE algorithm was used to evaluate differences in immune cell infiltration levels between high- and low-risk groups and to analyze immune-related functions to determine whether there were differences between individuals with different risk scores. To assess the responsiveness of patients in the high- and low-risk groups to immunotherapy, the Tumor Immune Dysfunction and Exclusion (TIDE) algorithm (http://tide.dfci.harvard.edu/login/) was applied. The expression levels of immune checkpoints in the high- and low-risk groups were analyzed to predict potential responsiveness to immunotherapy.

### Drug sensitivity assessment

To explore the clinical significance of the DRlncRNA-based prognostic model in drug therapy, the “oncoPredict” *R* package was used to analyze the half-maximal Inhibitory Concentration (IC50) of various chemotherapy drugs in the high-risk and low-risk groups. A total of 198 different drugs were included in the analysis, including cisplatin, camptothecin, docetaxel, and gemcitabine.

### Statistical analysis

Data processing was performed using *R* software (version 4.2.2) and Perl software (version 10.0.19044.2728). *R* software was also used for data visualization. Univariate and multivariate Cox regression analyses were conducted to evaluate the potential of the risk score as an independent prognostic indicator. ROC curves were used to assess the predictive accuracy of the prognostic model. Kaplan-Meier survival analysis was performed to compare OS and Progression-Free Survival (PFS) between the high-risk and low-risk groups. A *p-*value of less than 0.05 was considered statistically significant.

## Results

### Expression levels of the SLC7A11 gene in patients with HNSCC

**Fig. 1:**
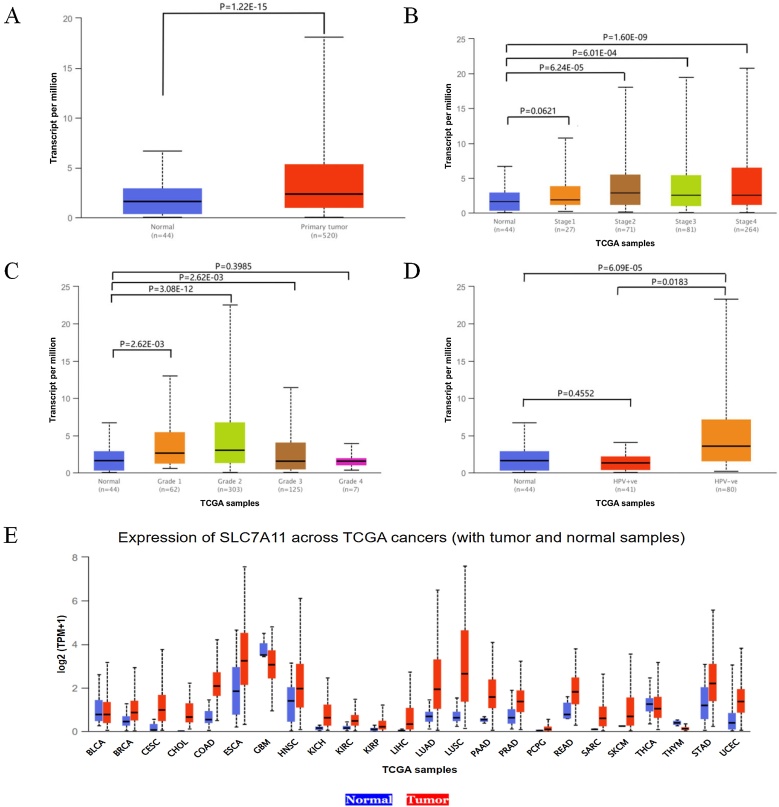
Expression of the SLC7A11 gene in HNSCC and pan-cancer analysis. (A) Expression levels of the SLC7A11 gene in normal tissues (*n* = 44) and primary HNSCC tumors (*n* = 520). (B) Expression of SLC7A11 in HNSCC based on cancer stages. (C) Expression of SLC7A11 in HNSCC based on tumor grades. (D) Expression of SLC7A11 in HNSCC based on HPV status. (E) Expression levels of SLC7A11 across different cancer types (including tumor and normal samples) in the TCGA database.

The expression levels of the SLC7A11 gene in HNSCC tissues were analyzed using the UALCAN database based on cancer stage, tumor grade, and HPV status. Compared to normal tissues, the expression of SLC7A11 was significantly upregulated in primary HNSCC patients (Fig. 1A). SLC7A11 expression was higher in stage 2, stage 3, and stage 4 HNSCC patients compared to normal tissues (Fig. 1B). Based on tumor grade, SLC7A11 expression was also elevated in patients with grade 1, grade 2, and grade 3 HNSCC compared to normal tissues (Fig. 1C). Regarding HPV status (P16 & ISH), the difference in SLC7A11 expression between normal samples and HPV-negative HNSCC patients was statistically significant (Fig. 1D). Further analysis of SLC7A11 expression across various cancer types revealed that its expression was higher in most tumor tissues compared to normal tissues (Fig. 1E).

### Data processing

**Fig. 2:**
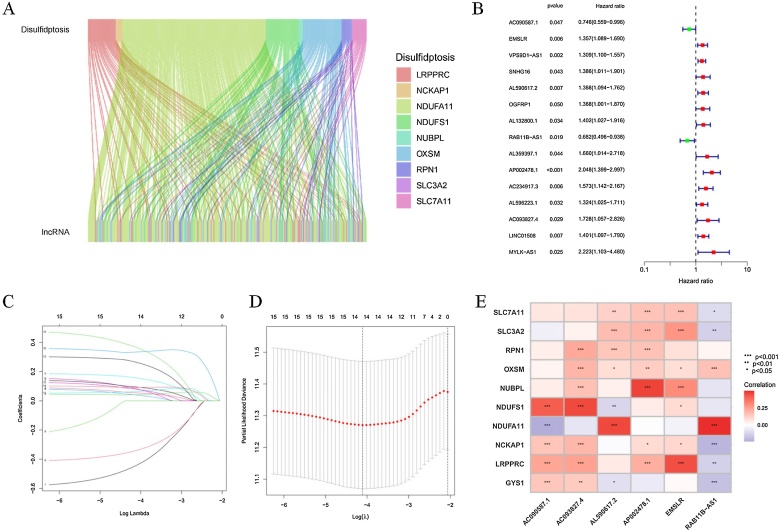
Identification of DRlncRNAs. (A) The Sankey diagram shows the co-expression relationships between disulfidptosis-related genes and 296 DRlncRNAs. (B) The forest plot displays the results of univariate Cox regression analysis for 15 prognostically differentially expressed DRlncRNAs. (C and D) Lasso-Cox regression analysis identified 14 DRlncRNAs. (E) The heatmap illustrates the relationships between disulfidptosis-related genes and six prognostically relevant DRlncRNAs; *p <  0.05, **p <  0.01, ***p <  0.001.

After obtaining the transcriptome data, we matched it with the annotation files from the GENCODE database and removed protein-coding genes from the TCGA-HNSCC dataset, ultimately identifying 16,876 lncRNAs. Subsequently, using Pearson correlation analysis, we identified 296 lncRNAs associated with disulfidptosis based on the expression data of disulfidptosis-related genes and lncRNAs in the TCGA-HNSCC cohort. A Sankey plot visualized the relationships between disulfidptosis-related genes and lncRNAs (Fig. 2A). Next, univariate Cox regression analysis was performed to evaluate the DRlncRNAs (*p* <  0.05). The 518 patients were divided into a training group (*n* = 259) and a validation group (*n* = 259). The clinical information of HNSCC patients is summarized in Table 1. The results showed no significant differences in clinical characteristics between the training and validation groups.

**Table 1:** Clinical information of 518 HNSCC patients in the TCGA database.

Covariates	Type	Total	Test	Train	*p*-value
Age	≤65	341 (65.83%)	169 (65.25%)	172 (66.41%)	0.853
	>65	177 (34.17%)	90 (34.75%)	87 (33.59%)	
Gender	Female	136 (26.25%)	69 (26.64%)	67 (25.87%)	0.9205
	Male	382 (73.75%)	190 (73.36%)	192 (74.13%)	
Grade	G1	62 (11.97%)	30 (11.58%)	32 (12.36%)	0.3143
	G2	303 (58.49%)	149 (57.53%)	154 (59.46%)	
	G3	124 (23.94%)	63 (24.32%)	61 (23.55%)	
	G4	7 (1.35%)	1 (0.39%)	6 (2.32%)	
	Unknow	22 (4.25%)	16 (6.18%)	6 (2.32%)	
Stage	Stage I	27 (5.21%)	13 (5.02%)	14 (5.41%)	0.9209
	Stage II	70 (13.51%)	34 (13.13%)	36 (13.9%)	
	Stage III	81 (15.64%)	39 (15.06%)	42 (16.22%)	
	Stage IV	265 (51.16%)	137 (52.9%)	128 (49.42%)	
	Unknow	75 (14.48%)	36 (13.9%)	39 (15.06%)	
T	T0	1 (0.9%)	1 (0.39%)	0 (0%)	0.8433
	T1	48 (9.27%)	26 (10.04%)	22 (8.49%)	
	T2	135 (26.06%)	68 (26.25%)	67 (25.87%)	
	T3	99 (19.11%)	48 (18.53%)	51 (19.69%)	
	T4	173 (33.4%)	88 (33.98%)	85 (32.82%)	
	Unknow	62 (11.97%)	28 (10.81%)	34 (13.13%)	
M	M0	184 (35.52%)	101 (39%)	83 (32.05%)	0.9263
	M1	1 (0.19%)	0 (0%)	1 (0.39%)	
	Unknow	333 (64.29%)	158 (61%)	175 (67.57%)	
N	IN	174 (33.59%)	87 (33.59%)	87 (33.59%)	0.9142
	N1	67 (12.93%)	36 (13.9%)	31 (11.97%)	
	NZ	169 (32.63%)	82 (31.66%)	87 (33.59%)	
	N3	8 (1.54%)	4 (1.54%)	4 (1.54%)	
	Unknow	100 (19.31%)	50 (19.31%)	50 (19.31%)	

### Identification of six disulfidptosis-related lncRNAs

Through univariate Cox regression analysis, 15 DRlncRNAs were identified (Fig. 2B). Subsequently, 14 DRlncRNAs were filtered using Lasso-Cox regression, and the changing trajectory of regression coefficients as well as the results of cross-validation were analyzed (Fig. 2C and D). Next, multivariate Cox regression analysis was performed, and six DRlncRNAs associated with survival were ultimately selected to construct a risk scoring model. A heatmap illustrates the correlation between disulfidptosis-related genes and DRlncRNAs (Fig. 2E).

### Construction of the prognostic model

**Fig. 3:**
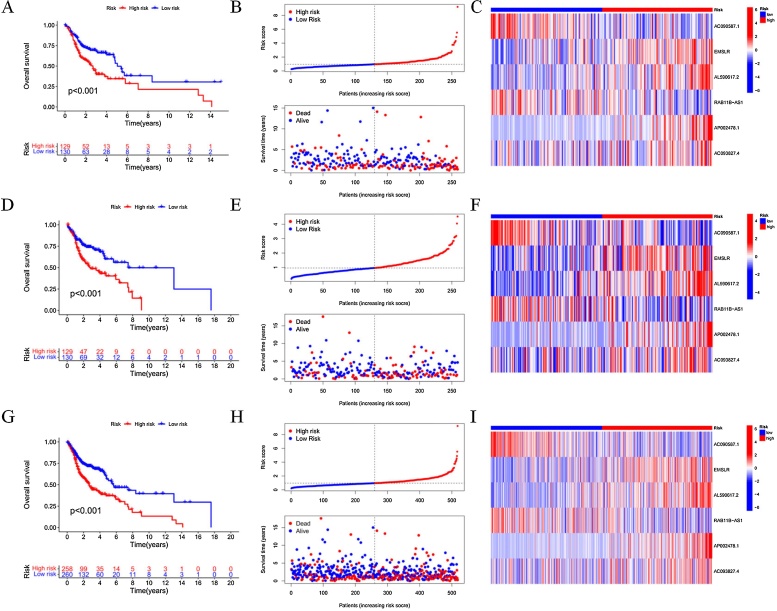
Construction of the DRlncRNAs prognostic risk model. (A) Kaplan-Meier survival curve analysis for the training cohort. (B) Risk score distribution and survival status in the training cohort. (C) Heatmap of prognostic biomarkers in the training cohort. (D) Kaplan-Meier survival curve analysis for the testing cohort. (E)Risk score distribution and survival status in the testing cohort. (F) Heatmap of prognostic biomarkers in the testing cohort. (G) Kaplan-Meier survival curve analysis for the entire cohort. (H) Risk score distribution and survival status in the entire cohort. (I) Heatmap of prognostic biomarkers in the entire cohort.

The formula for calculating the prognostic model risk score is as follows: (−0.486528812331369 × ExpressionAC090587.1) + (0.189409569570024 × ExpressionEMSLR) + (0.284273817027948 × ExpressionAL590617.2) + (−0.416825741160816 × ExpressionRAB11B-AS1) + (0.43473763351908 × ExpressionAP002478.1) + (0.55499168417481 × ExpressionAC093827.4). Kaplan-Meier curve analysis showed that patients in the high-risk group had significantly shorter OS compared to those in the low-risk group in the training set (*p* <  0.001; Fig. 3A). Fig. 3B illustrates the survival status and risk scores of patients in the training set, showing that as the risk score increased, survival time decreased, and the number of deaths increased. The heatmap (Fig. 3C) displays the expression levels of the six DRlncRNAs in the high- and low-risk groups. In the testing set, the OS difference between the high- and low-risk groups was also statistically significant (*p* <  0.001; Fig. 3D‒F). Similarly, in the entire cohort, the OS difference between the high- and low-risk groups was also statistically significant (*p* <  0.001; Fig. 3G‒I).

We further analyzed and developed a prognostic prediction model specifically for HPV-negative HNSCC patients. Based on ISH and P16 testing results, 84 HPV-negative samples were selected for model construction. The limited sample size was primarily due to the lack of clear HPV status annotation for most samples. Using the constructed prognostic model, we found that both OS and PFS showed statistically significant differences between the high- and low-risk groups. Moreover, the predictive performance of the model was validated through ROC curve analysis, demonstrating good performance (Fig. S1 in Supplementary material). Nevertheless, we also acknowledge that the relatively small sample size may affect the stability and generalizability of the model. Therefore, future studies incorporating larger sample sizes for further validation and optimization are expected to enhance the predictive efficiency and clinical applicability of the model.

### Validation of the accuracy of the DRlncRNA prognostic risk model

**Fig. 4:**
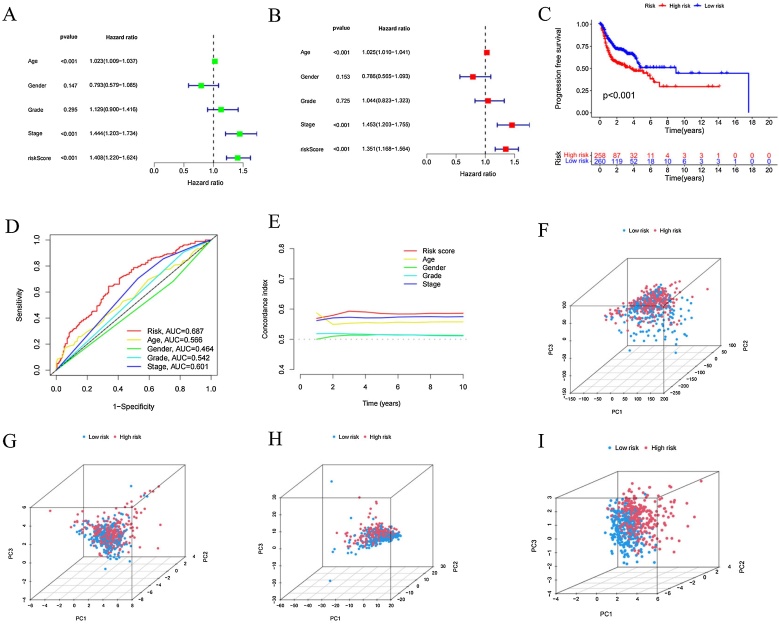
Independent prognostic analysis of overall survival in HNSCC. (A) Univariate cox regression analysis of DRlncRNA characteristics in the full sample. (B) Multivariate Cox regression analysis of DRlncRNA characteristics. (C) Kaplan-Meier curve for PFS. (D) ROC curve comparing the AUC values of the risk score and clinical characteristics for prognostic accuracy. (E) Concordance index comparing the prognostic accuracy of the risk score and clinical factors. PCA of the high-risk and low-risk groups based on: (F) All genes, (G) Disulfidptosis-related genes, (H) DRlncRNAs, (I) Expression characteristics of the risk model constructed by six DRlncRNAs in the TCGA cohort.

To determine whether the six DRlncRNAs have significant predictive value for HNSCC patients, univariate and multivariate Cox regression analyses were performed (HR = 1.408, 95% CI: 1.220–1.624, *p* <  0.001; HR = 1.351, 95% CI: 1.168–1.564, *p* <  0.001). As shown in Fig. 4A and B, age and clinical stage were associated with the risk characteristics of the six DRlncRNAs. Further analysis revealed a significant difference in PFS between the high-risk and low-risk groups (*p* < 0.01, Fig. 4C). The predictive ability of the prognostic model was validated using ROC curves, where the AUC value of the risk score was significantly higher than that of other clinical parameters (Fig. 4D). Over time, the concordance index of the risk score consistently outperformed any other clinical factor, suggesting that the risk score may serve as a more reliable predictor of outcomes in HNSCC patients (Fig. 4E). Principal Component Analysis (PCA) indicated differences between the high-risk and low-risk groups, with HNSCC patients successfully divided into two distinct groups (Fig. 4F‒I).

### Development and evaluation of a disulfidptosis-related lncRNA predictive nomogram

**Fig. 5:**
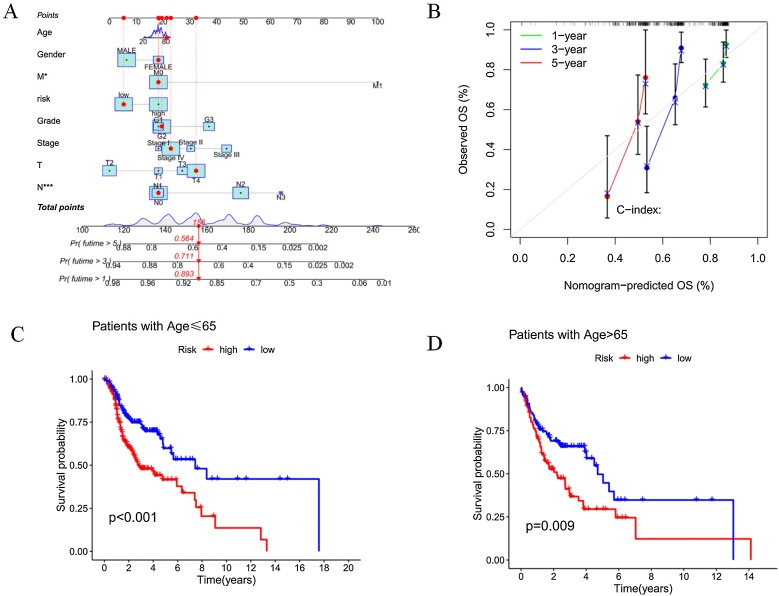
Construction and evaluation of the nomogram based on DRlncRNAs. (A) Nomogram for predicting 1-year, 3-year, and 5-year overall survival in HNSCC patients. (B) Calibration curves assessing the accuracy of the nomogram model. (C) Kaplan-Meier survival curve for patients aged ≤ 65 years. (D) Kaplan-Meier survival curve for patients aged > 65-years.

A customized OS prediction model was developed for HNSCC patients based on factors such as gender, age, grade, risk score, TNM stage, and pathological stage. The nomogram was designed to predict 1-year, 3-year, and 5-year OS, evaluating patients based on risk levels and other factors (Fig. 5A). The calibration plot demonstrated the accuracy of the nomogram predictions (Fig. 5B). Furthermore, the relationship between DRlncRNAs and clinical characteristics was investigated, and the results indicated that the prognostic model performed well in predicting outcomes across patients of different age groups (Fig. 5C and D).

### Gene set enrichment analysis and pathway enrichment analysis

**Fig. 6:**
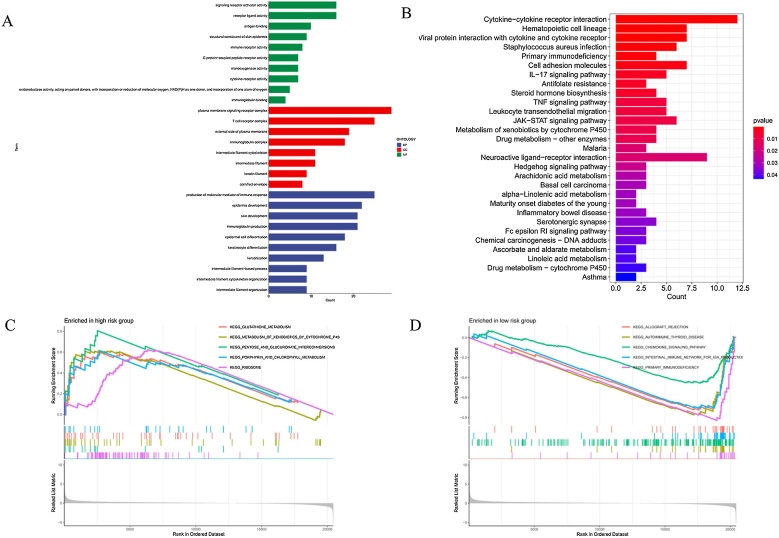
Functional enrichment analysis. (A) GO enrichment analysis. (B) KEGG enrichment analysis. (C) GSEA analysis showing the top 5 significantly enriched pathways in the high-risk group. (D) GSEA analysis showing the top 5 significantly enriched pathways in the low-risk group.

GO and KEGG enrichment analyses were performed to investigate the biological processes and pathways of differentially expressed genes between the high-risk and low-risk groups. GO enrichment analysis revealed that the Biological Process, Cellular Component, and Molecular Function categories were mainly associated with signaling receptor activator activity, plasma membrane signaling receptor complex, and production of molecular mediators of immune response (Fig. 6A). In the KEGG pathway category, pathways with significant gene enrichment included hematopoietic cell lineage, viral protein interaction with cytokines and cytokine-cytokine receptor interaction (Fig. 6B). GSEA analysis indicated that the KEGG pathway enriched in the high-risk group was the pentose and glucuronate interconversion pathway, whereas the pathway enriched in the low-risk group was primary immunodeficiency (Fig. 6C and D).

### TMB of the disulfidptosis-related lncRNA risk model serves as a prognostic marker for HNSCC

**Fig. 7:**
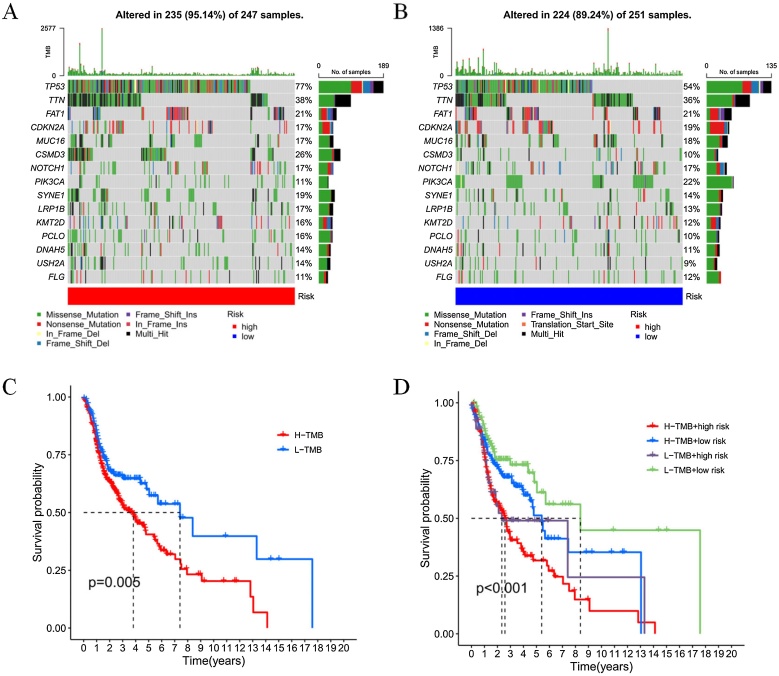
TMB characteristics in the risk model of DRlncRNAs. (A) Waterfall plot of the top 15 most frequently mutated genes in the high-risk group. (B) Waterfall plot of the top 15 most frequently mutated genes in the low-risk group. (C) Survival analysis curve comparing the high-TMB and low-TMB groups. (D) Survival analysis curve of TMB combined with risk scores across different subgroups.

To determine the mutation frequency of genomic genes corresponding to the risk model, somatic mutation data were collected. Based on these data, waterfall plots of the top 15 mutated genes were generated (Fig. 7A and B). TP53 mutations were the most common in both the high-risk and low-risk groups. Each HNSCC patient was classified into either a high-TMB group or a low-TMB group based on their TMB values. Kaplan-Meier curve analysis revealed that patients in the low-TMB group had significantly longer survival times compared to those in the high-TMB group (Fig. 7C). Subgroup survival analysis further indicated that patients in the low-TMB combined with low-risk group had markedly better survival outcomes than those in the high-TMB combined with high-risk group (Fig. 7D).

### Assessment of tumor-infiltrating immune cells and immunotherapy

**Fig. 8:**
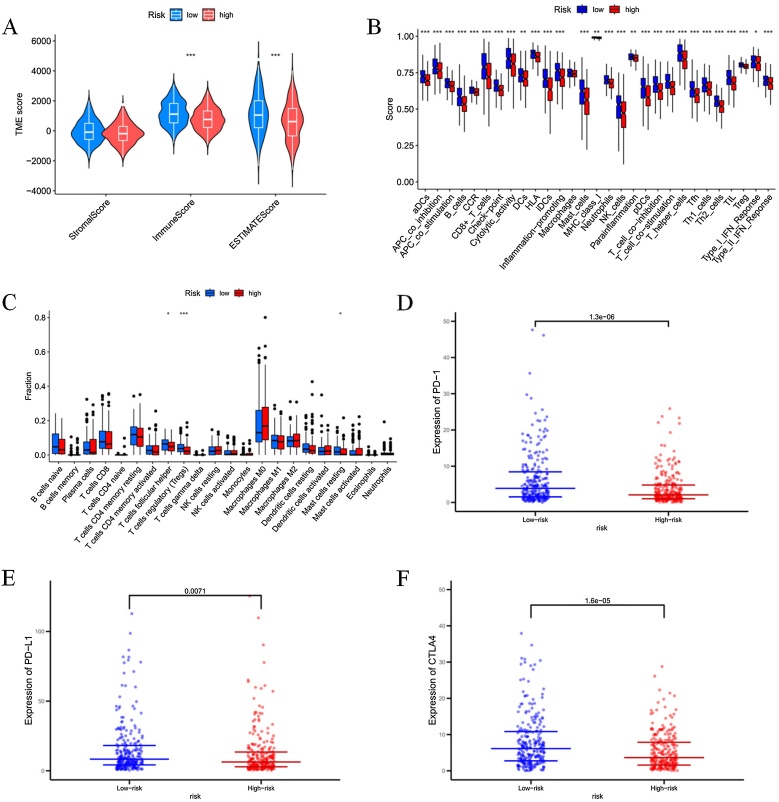
Assessment of tumor-infiltrating immune cells and immunotherapy. (A) Stromal score, immune score, and ESTIMATE score in the low-risk and high-risk groups of HNSCC patients. (B) Comparison of immune functions between the high-risk and low-risk groups. (C) Immune cell infiltration in the high-risk and low-risk groups. (D–F) Relative expression levels of PD-1, PD-L1, and CTLA-4 in the high- and low-risk groups (*p <  0.05, **p <  0.01, ***p <  0.001).

The tumor microenvironment was analyzed using the *R* package “estimate”, and the immune cell scores in the tumor microenvironment were significantly higher in the low-risk group compared to the high-risk group (Fig. 8A). Analysis of immune cell infiltration showed that follicular helper T-cells, regulatory T-cells (Tregs), and resting mast cells were more abundant in the low-risk group than in the high-risk group (Fig. 8C). Additionally, differences in immune functions were observed between the high- and low-risk groups (Fig. 8B). Further analysis of the expression of key immune-related genes revealed that the expression levels of three immune checkpoints (PD-1, PD-L1, and CTLA-4) were significantly higher in the low-risk group compared to the high-risk group (Fig. 8D–F). These results suggest that patients in the low-risk group may have a higher potential responsiveness to Immune Checkpoint Inhibitor (ICI) therapy.

### Drug sensitivity

**Fig. 9:**
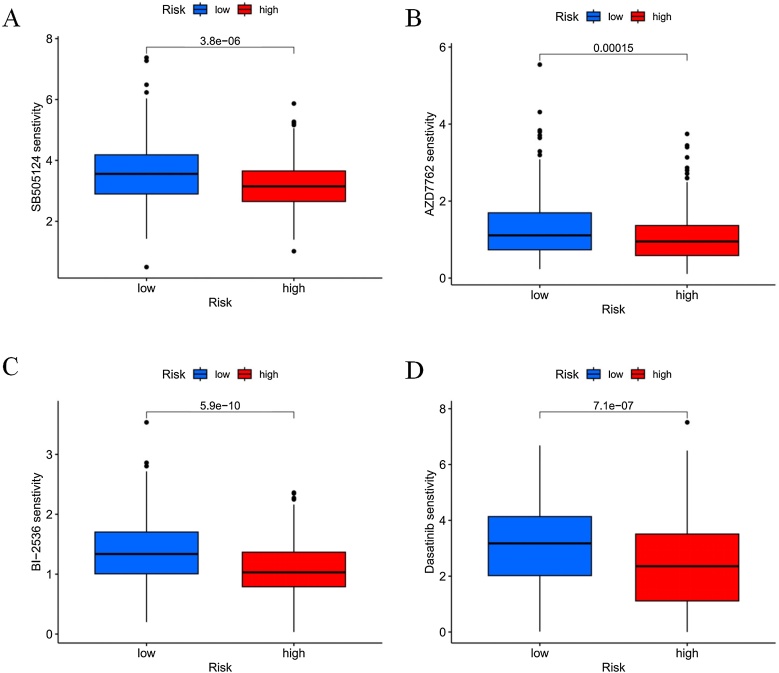
Potential sensitive drugs for the high-risk group.

The relationship between the HNSCC risk score and drug IC50 was investigated to explore the potential application value of DRlncRNAs in the personalized treatment of HNSCC. A statistically significant difference in the sensitivity of 99 anti-cancer drugs was observed between the high-risk and low-risk groups (*p* <  0.05). Meanwhile, four drugs (SB505124, AZD7762, BI-2536, and Dasatinib) exhibited lower IC50 values in the high-risk group, suggesting their potential application value in this group (Fig. 9).

## Discussion

To meet the demands of personalized precision medicine, researchers are dedicated to identifying biomarkers that can predict the prognosis and treatment response of HNSCC. Recent studies have demonstrated that gene signatures related to ubiquitination,^16^ oxidative stress,^17^ and apoptosis have been utilized to assess the prognosis and treatment response of HNSCC patients.^18^ Disulfidptosis, a newly discovered mode of cell death, has been studied in lung and colon cancer, revealing an association between disulfidptosis-related genes and various immune cells, suggesting its potential role in shaping the tumor microenvironment.^19^, ^20^ However, the specific role of disulfidptosis in the prognosis of HNSCC remains to be explored, particularly in HPV-negative subgroups, where research is still lacking.

In this study, we constructed a risk prediction model comprising six DRlncRNAs and stratified HNSCC patients into high-risk and low-risk groups based on calculated risk scores. Survival analysis showed that both OS and PFS were significantly lower in the high-risk group than in the low-risk group. The accuracy and stability of this model were further validated through ROC curves and calibration curves. However, although this study constructed a prognostic model based on DRlncRNAs, the specific molecular mechanisms and signaling pathways involved in the occurrence and progression of HNSCC remain unclear and require further investigation and validation.

To uncover the potential biological differences between the risk groups, we conducted a systematic analysis of TMB, Tumor Microenvironment (TME), and immune cell infiltration characteristics. TMB analysis revealed that TP53 mutations were the most common mutations in both high- and low-risk groups. TCGA data analysis indicated that smoking-associated HNSCC exhibited a higher frequency of TP53 and CDKN2A inactivating mutations, along with copy number amplifications in 3q26/28 and 11q13/22, whereas HPV-positive HNSCC was predominantly characterized by PIK3CA helical domain mutations.^21^ Although no significant difference was observed in stromal scores between the high- and low-risk groups, the immune cell infiltration levels and immune function scores were significantly higher in the low-risk group. This suggests that the immune microenvironment may play a crucial role in HNSCC prognosis. Further analysis revealed that the infiltration levels of follicular helper T-cells (Tfh cells), regulatory T-cells (Tregs), and resting mast cells were higher in the low-risk group. Previous studies have shown that a high abundance of Tfh cells is associated with better survival outcomes in HNSCC patients, potentially by promoting the recruitment and activation of other immune cells, thereby enhancing anti-tumor immune responses.^22^ Additionally, cell death can induce the release of immunogenic substances, activating immune cells and boosting their anti-tumor activity.^23^ For example, Peroxisome Proliferator-Activated Receptor gamma (PPARγ) inhibitors can induce disulfidptosis in oral squamous carcinoma cells by upregulating SLC7A11, thereby promoting the recruitment of classical Dendritic Cells (cDCs) and CD8^+^ T-cells into the tumor microenvironment, ultimately suppressing tumor progression.^24^ These findings suggest that reduced immune cell infiltration may be a key factor contributing to the poor prognosis of high-risk patients.

With neoadjuvant immunotherapy now approved as a first-line treatment for refractory Recurrent/Metastatic HNSCC (R/M-HNSCC), immunotherapy has reshaped the treatment landscape of HNSCC.^25^ However, in clinical practice, its overall response rate remains low (only 14%–32%), and its efficacy is difficult to predict.^26^ Our study found that PD-1, PD-L1, and CTLA-4 expression levels were significantly higher in the low-risk group than in the high-risk group. Activation of the PD-1/PD-L1 axis can trigger a series of immunosuppressive responses, including T-cell metabolic reprogramming, effector T-cell dysfunction, and T-cell exhaustion.^27^ Previous studies have shown that high PD-L1 expression in HNSCC tissues is associated with distant metastasis and poor prognosis, regardless of the tumor's primary site.^28^ However, other studies suggest that high PD-1/PD-L1 expression may indicate better prognosis, particularly in HPV-positive patients, likely due to the direct correlation between p16 protein expression and PD-1/PD-L1 levels. Therefore, the better prognosis observed in the low-risk group may be linked to their greater responsiveness to immunotherapy.^29^, ^30^, ^31^ Given that HPV infection may influence PD-1/PD-L1 expression, future studies should further differentiate subgroups based on HPV status and conduct in-depth analyses of immune cell infiltration and immune-related gene expression patterns.

To address the clinical needs of high-risk patients with poor prognosis, we identified four potential therapeutic agents (SB505124, AZD7762, BI-2536, and Dasatinib) that may be effective in this subgroup. SB505124, an inhibitor of the TGF-β signaling pathway, can reduce the expression of matrix metalloproteinase-9 in HNSCC cells.^32^ AZD7762, a Checkpoint Kinase-1 (Chk1) inhibitor, can enhance cisplatin sensitivity in p53-deficient HNSCC cells by inducing mitotic cell death.^33^ BI-2536, primarily targeting the ATP-binding domain of Polo-Like Kinase-1 (PLK1),^34^ has been shown to reduce clonogenic, invasive, and migratory abilities of HNSCC cells, particularly when combined with Erastin (a ferroptosis inducer).^35^ Dasatinib, a multi-targeted oral tyrosine kinase inhibitor, is primarily used to treat imatinib-resistant chronic myeloid leukemia but has shown limited efficacy in HNSCC clinical trials.^36^, ^37^, ^38^ Although our study predicts that these four drugs may have therapeutic potential in high-risk patients, their clinical applicability requires further validation.

Despite its contributions, this study has several limitations. First, the reliability of the proposed model has yet to be validated in independent external datasets or clinical cohorts. Second, the prognostic value of DRlncRNAs in HPV-positive patients has not been established, and future studies should explore DRlncRNA-based prognostic models in different HPV subgroups. Additionally, this study is primarily based on bioinformatics analyses, lacking experimental validation. Future research should conduct in vitro and in vivo experiments to elucidate the specific functions of DRlncRNAs in HNSCC.

## Conclusion

This study constructed a prognostic model consisting of six DRlncRNAs, which demonstrated prognostic value in HNSCC. Furthermore, a prognostic model specifically targeting HPV-negative HNSCC was developed. These findings provide potential new insights for prognosis prediction and immune therapy response in HNSCC.

## Funding

This work has been supported by National Health and Health Commission Key Laboratory Fund Project “Study on the immune escape mechanism of EBV-associated gastric cancer based on the EBV-miR-BART5-enhancer-PDL1/PD1 axis” (NHCDP2022005); Natural Science Foundation of Gansu Province Project “EBV virus-derived miRNA-BART18-3 P participates in gastric cancer development through the adipose de novo synthesis pathway Participation in Gastric Cancer Development” (24JRRA586); Gansu Provincial Science and Technology Department Joint Fund Project “Key Molecular Mechanisms and Clinical Diagnosis and Treatment of Colorectal Cancer Occurrence, Recurrence, and Metastasis” (23JRRA1545); Gansu Provincial People's Hospital 2022 Master's Degree/Postdoctoral Fund Project “HPV16-miR-1 Promotes Tongue Squamous Carcinoma Proliferation and Metastasis via Regulating CERK/PI3K/AKT Axis Study on the role of HPV16-miR-1 in promoting the proliferation and metastasis of tongue squamous carcinoma through regulating the CERK/PI3K/AKT axis” (22GSSYD-44).

## Declaration of competing interest

The authors declare no conflicts of interest.
