# Supplementary material for: Disulfidptosis ‒ related lncRNAs are biomarkers of prognosis and immune response in Head and Neck Squamous Cell Carcinoma
Source: Braz J Otorhinolaryngol. 2025 May 15;91(5):101625. doi: 10.1016/j.bjorl.2025.101625 (PMC12144424; doi:10.1016/j.bjorl.2025.101625)

**BJORL-D-24-00336_ Supplementary Material**

**Supplementary Figure 1** Prognostic model for HPV-negative HNSCC and analysis of its predictive performance. (A) Forest plot showing the univariate Cox regression analysis results of the 5 DRlncRNAs. (B‒C) Lasso-Cox regression analysis for selecting the 5 DRlncRNAs. (D) Kaplan-Meier curve for OS. (E) Kaplan-Meier curve for PFS. (F) ROC curves comparing the prognostic accuracy (AUC values) of the risk score and clinical characteristics.


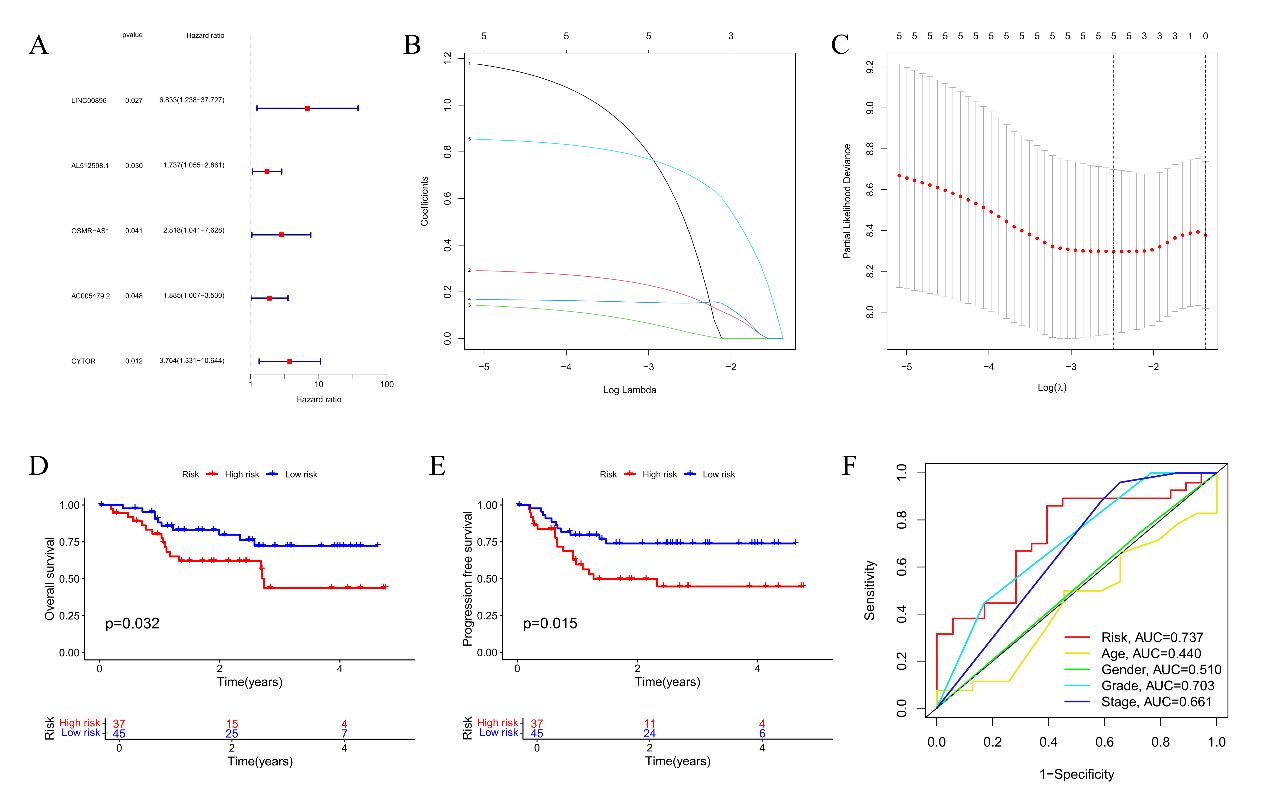

Supplement: Supplementary file 1 [file mmc1.docx]
